# Supplementary material for: Pyruvate Dehydrogenase Kinase 1 inhibition mediated oxidative phosphorylation enhancement in cartilage promotes osteoarthritis progression
Source: BMC Musculoskelet Disord. 2023 Jul 20;24:597. doi: 10.1186/s12891-023-06585-6 (PMC10357736; doi:10.1186/s12891-023-06585-6)
Supplement: Supplementary file 1 — Additional file 1. [file 12891_2023_6585_MOESM1_ESM.zip › MF.Gsea.1653627316242/GOMF_PROTEIN_SERINE_KINASE_ACTIVITY.html]

Details for gene set GOMF\_PROTEIN\_SERINE\_KINASE\_ACTIVITY[GSEA]

|  || Dataset | 55457\_collapsed\_to\_symbols.cls.cls #OA\_versus\_normal.cls.cls #OA\_versus\_normal\_repos |
| Phenotype | cls.cls#OA\_versus\_normal\_repos |
| Upregulated in class | normal |
| GeneSet | GOMF\_PROTEIN\_SERINE\_KINASE\_ACTIVITY |
| Enrichment Score (ES) | -0.35301006 |
| Normalized Enrichment Score (NES) | -1.7847444 |
| Nominal p-value | 0.0 |
| FDR q-value | 0.07784142 |
| FWER p-Value | 0.604 |
Table: GSEA Results Summary

  

Fig 1: Enrichment plot: GOMF\_PROTEIN\_SERINE\_KINASE\_ACTIVITY      
 Profile of the Running ES Score & Positions of GeneSet Members on the Rank Ordered List

  

| SYMBOL | TITLE | RANK IN GENE LIST | RANK METRIC SCORE | RUNNING ES | CORE ENRICHMENT || 1 | DYRK4 | dual specificity tyrosine phosphorylation regulated kinase 4 [Source:HGNC Symbol;Acc:HGNC:3095] | 6 | 1.217 | 0.0127 | No |
| 2 | STK16 | serine/threonine kinase 16 [Source:HGNC Symbol;Acc:HGNC:11394] | 318 | 0.738 | -0.0041 | No |
| 3 | SRPK3 | SRSF protein kinase 3 [Source:HGNC Symbol;Acc:HGNC:11402] | 329 | 0.734 | 0.0031 | No |
| 4 | SGK3 | serum/glucocorticoid regulated kinase family member 3 [Source:HGNC Symbol;Acc:HGNC:10812] | 609 | 0.620 | -0.0124 | No |
| 5 | CSNK1G3 | casein kinase 1 gamma 3 [Source:HGNC Symbol;Acc:HGNC:2456] | 705 | 0.592 | -0.0135 | No |
| 6 | MAPK9 | mitogen-activated protein kinase 9 [Source:HGNC Symbol;Acc:HGNC:6886] | 721 | 0.588 | -0.0084 | No |
| 7 | MAPK3 | mitogen-activated protein kinase 3 [Source:HGNC Symbol;Acc:HGNC:6877] | 757 | 0.579 | -0.0049 | No |
| 8 | PRKACB | protein kinase cAMP-activated catalytic subunit beta [Source:HGNC Symbol;Acc:HGNC:9381] | 771 | 0.576 | 0.0003 | No |
| 9 | CDK7 | cyclin dependent kinase 7 [Source:HGNC Symbol;Acc:HGNC:1778] | 834 | 0.560 | 0.0014 | No |
| 10 | CAMK2A | calcium/calmodulin dependent protein kinase II alpha [Source:HGNC Symbol;Acc:HGNC:1460] | 916 | 0.538 | 0.0008 | No |
| 11 | NEK4 | NIMA related kinase 4 [Source:HGNC Symbol;Acc:HGNC:11399] | 992 | 0.519 | 0.0004 | No |
| 12 | TNNI3K | TNNI3 interacting kinase [Source:HGNC Symbol;Acc:HGNC:19661] | 1166 | 0.483 | -0.0081 | No |
| 13 | AKT1 | AKT serine/threonine kinase 1 [Source:HGNC Symbol;Acc:HGNC:391] | 1192 | 0.478 | -0.0049 | No |
| 14 | ILK | integrin linked kinase [Source:HGNC Symbol;Acc:HGNC:6040] | 1286 | 0.460 | -0.0073 | No |
| 15 | CDK4 | cyclin dependent kinase 4 [Source:HGNC Symbol;Acc:HGNC:1773] | 1375 | 0.447 | -0.0095 | No |
| 16 | MAP2K5 | mitogen-activated protein kinase kinase 5 [Source:HGNC Symbol;Acc:HGNC:6845] | 1391 | 0.444 | -0.0059 | No |
| 17 | AURKB | aurora kinase B [Source:HGNC Symbol;Acc:HGNC:11390] | 1407 | 0.442 | -0.0023 | No |
| 18 | PIK3R4 | phosphoinositide-3-kinase regulatory subunit 4 [Source:HGNC Symbol;Acc:HGNC:8982] | 1424 | 0.439 | 0.0012 | No |
| 19 | EIF2AK1 | eukaryotic translation initiation factor 2 alpha kinase 1 [Source:HGNC Symbol;Acc:HGNC:24921] | 1442 | 0.437 | 0.0045 | No |
| 20 | CDK14 | cyclin dependent kinase 14 [Source:HGNC Symbol;Acc:HGNC:8883] | 1549 | 0.420 | 0.0007 | No |
| 21 | MAP3K7 | mitogen-activated protein kinase kinase kinase 7 [Source:HGNC Symbol;Acc:HGNC:6859] | 1701 | 0.399 | -0.0070 | No |
| 22 | TSSK2 | testis specific serine kinase 2 [Source:HGNC Symbol;Acc:HGNC:11401] | 1968 | 0.364 | -0.0242 | No |
| 23 | CDK16 | cyclin dependent kinase 16 [Source:HGNC Symbol;Acc:HGNC:8749] | 1975 | 0.363 | -0.0207 | No |
| 24 | MYO3A | myosin IIIA [Source:HGNC Symbol;Acc:HGNC:7601] | 2032 | 0.358 | -0.0213 | No |
| 25 | PINK1 | PTEN induced kinase 1 [Source:HGNC Symbol;Acc:HGNC:14581] | 2097 | 0.350 | -0.0226 | No |
| 26 | TNIK | TRAF2 and NCK interacting kinase [Source:HGNC Symbol;Acc:HGNC:30765] | 2133 | 0.346 | -0.0217 | No |
| 27 | CILK1 | ciliogenesis associated kinase 1 [Source:HGNC Symbol;Acc:HGNC:21219] | 2135 | 0.346 | -0.0180 | No |
| 28 | STK25 | serine/threonine kinase 25 [Source:HGNC Symbol;Acc:HGNC:11404] | 2152 | 0.344 | -0.0156 | No |
| 29 | MAP2K4 | mitogen-activated protein kinase kinase 4 [Source:HGNC Symbol;Acc:HGNC:6844] | 2214 | 0.337 | -0.0168 | No |
| 30 | PRKCD | protein kinase C delta [Source:HGNC Symbol;Acc:HGNC:9399] | 2237 | 0.335 | -0.0149 | No |
| 31 | CASK | calcium/calmodulin dependent serine protein kinase [Source:HGNC Symbol;Acc:HGNC:1497] | 2316 | 0.324 | -0.0176 | No |
| 32 | MAPK14 | mitogen-activated protein kinase 14 [Source:HGNC Symbol;Acc:HGNC:6876] | 2352 | 0.320 | -0.0169 | No |
| 33 | BUB1 | BUB1 mitotic checkpoint serine/threonine kinase [Source:HGNC Symbol;Acc:HGNC:1148] | 2388 | 0.315 | -0.0163 | No |
| 34 | TTN | titin [Source:HGNC Symbol;Acc:HGNC:12403] | 2492 | 0.303 | -0.0212 | No |
| 35 | CAMKK2 | calcium/calmodulin dependent protein kinase kinase 2 [Source:HGNC Symbol;Acc:HGNC:1470] | 2497 | 0.303 | -0.0182 | No |
| 36 | CHEK2 | checkpoint kinase 2 [Source:HGNC Symbol;Acc:HGNC:16627] | 2533 | 0.299 | -0.0178 | No |
| 37 | PKN1 | protein kinase N1 [Source:HGNC Symbol;Acc:HGNC:9405] | 2540 | 0.298 | -0.0150 | No |
| 38 | MAP4K2 | mitogen-activated protein kinase kinase kinase kinase 2 [Source:HGNC Symbol;Acc:HGNC:6864] | 2543 | 0.298 | -0.0120 | No |
| 39 | MAPK13 | mitogen-activated protein kinase 13 [Source:HGNC Symbol;Acc:HGNC:6875] | 2544 | 0.298 | -0.0088 | No |
| 40 | PRKCZ | protein kinase C zeta [Source:HGNC Symbol;Acc:HGNC:9412] | 2760 | 0.274 | -0.0229 | No |
| 41 | ULK2 | unc-51 like autophagy activating kinase 2 [Source:HGNC Symbol;Acc:HGNC:13480] | 2796 | 0.270 | -0.0227 | No |
| 42 | CAMK2G | calcium/calmodulin dependent protein kinase II gamma [Source:HGNC Symbol;Acc:HGNC:1463] | 2900 | 0.261 | -0.0281 | No |
| 43 | CHEK1 | checkpoint kinase 1 [Source:HGNC Symbol;Acc:HGNC:1925] | 3173 | 0.233 | -0.0471 | No |
| 44 | MAP4K1 | mitogen-activated protein kinase kinase kinase kinase 1 [Source:HGNC Symbol;Acc:HGNC:6863] | 3328 | 0.219 | -0.0570 | No |
| 45 | RPS6KA1 | ribosomal protein S6 kinase A1 [Source:HGNC Symbol;Acc:HGNC:10430] | 3389 | 0.213 | -0.0595 | No |
| 46 | CDK5 | cyclin dependent kinase 5 [Source:HGNC Symbol;Acc:HGNC:1774] | 3400 | 0.211 | -0.0580 | No |
| 47 | NEK9 | NIMA related kinase 9 [Source:HGNC Symbol;Acc:HGNC:18591] | 3444 | 0.207 | -0.0591 | No |
| 48 | MAPKAPK3 | MAPK activated protein kinase 3 [Source:HGNC Symbol;Acc:HGNC:6888] | 3483 | 0.204 | -0.0600 | No |
| 49 | PLK1 | polo like kinase 1 [Source:HGNC Symbol;Acc:HGNC:9077] | 3556 | 0.198 | -0.0635 | No |
| 50 | STK19 | serine/threonine kinase 19 [Source:HGNC Symbol;Acc:HGNC:11398] | 3585 | 0.195 | -0.0637 | No |
| 51 | PASK | PAS domain containing serine/threonine kinase [Source:HGNC Symbol;Acc:HGNC:17270] | 3604 | 0.193 | -0.0630 | No |
| 52 | RIOK2 | RIO kinase 2 [Source:HGNC Symbol;Acc:HGNC:18999] | 3615 | 0.192 | -0.0617 | No |
| 53 | PIM2 | "Pim-2 proto-oncogene, serine/threonine kinase [Source:HGNC Symbol;Acc:HGNC:8987]" | 3623 | 0.192 | -0.0602 | No |
| 54 | PRKCQ | protein kinase C theta [Source:HGNC Symbol;Acc:HGNC:9410] | 3658 | 0.189 | -0.0609 | No |
| 55 | CAMK4 | calcium/calmodulin dependent protein kinase IV [Source:HGNC Symbol;Acc:HGNC:1464] | 3664 | 0.189 | -0.0592 | No |
| 56 | RPS6KA4 | ribosomal protein S6 kinase A4 [Source:HGNC Symbol;Acc:HGNC:10433] | 3702 | 0.185 | -0.0602 | No |
| 57 | PRKCA | protein kinase C alpha [Source:HGNC Symbol;Acc:HGNC:9393] | 3728 | 0.183 | -0.0602 | No |
| 58 | CDK1 | cyclin dependent kinase 1 [Source:HGNC Symbol;Acc:HGNC:1722] | 3744 | 0.182 | -0.0594 | No |
| 59 | PAK1 | p21 (RAC1) activated kinase 1 [Source:HGNC Symbol;Acc:HGNC:8590] | 3861 | 0.169 | -0.0668 | No |
| 60 | HUNK | hormonally up-regulated Neu-associated kinase [Source:HGNC Symbol;Acc:HGNC:13326] | 3996 | 0.156 | -0.0757 | No |
| 61 | PRKD1 | protein kinase D1 [Source:HGNC Symbol;Acc:HGNC:9407] | 4123 | 0.144 | -0.0842 | No |
| 62 | VRK1 | VRK serine/threonine kinase 1 [Source:HGNC Symbol;Acc:HGNC:12718] | 4193 | 0.139 | -0.0881 | No |
| 63 | PSKH1 | protein serine kinase H1 [Source:HGNC Symbol;Acc:HGNC:9529] | 4199 | 0.138 | -0.0871 | No |
| 64 | RIOK3 | RIO kinase 3 [Source:HGNC Symbol;Acc:HGNC:11451] | 4206 | 0.137 | -0.0860 | No |
| 65 | NEK11 | NIMA related kinase 11 [Source:HGNC Symbol;Acc:HGNC:18593] | 4290 | 0.129 | -0.0912 | No |
| 66 | MAPK7 | mitogen-activated protein kinase 7 [Source:HGNC Symbol;Acc:HGNC:6880] | 4396 | 0.121 | -0.0983 | No |
| 67 | BUB1B | BUB1 mitotic checkpoint serine/threonine kinase B [Source:HGNC Symbol;Acc:HGNC:1149] | 4454 | 0.117 | -0.1015 | No |
| 68 | PBK | PDZ binding kinase [Source:HGNC Symbol;Acc:HGNC:18282] | 4459 | 0.117 | -0.1006 | No |
| 69 | CSNK1D | casein kinase 1 delta [Source:HGNC Symbol;Acc:HGNC:2452] | 4529 | 0.111 | -0.1049 | No |
| 70 | TAOK3 | TAO kinase 3 [Source:HGNC Symbol;Acc:HGNC:18133] | 4633 | 0.101 | -0.1119 | No |
| 71 | RIPK2 | receptor interacting serine/threonine kinase 2 [Source:HGNC Symbol;Acc:HGNC:10020] | 4848 | 0.083 | -0.1280 | No |
| 72 | AURKA | aurora kinase A [Source:HGNC Symbol;Acc:HGNC:11393] | 4916 | 0.079 | -0.1325 | No |
| 73 | VRK2 | VRK serine/threonine kinase 2 [Source:HGNC Symbol;Acc:HGNC:12719] | 4918 | 0.078 | -0.1317 | No |
| 74 | TESK2 | testis associated actin remodelling kinase 2 [Source:HGNC Symbol;Acc:HGNC:11732] | 4947 | 0.076 | -0.1331 | No |
| 75 | NUAK2 | NUAK family kinase 2 [Source:HGNC Symbol;Acc:HGNC:29558] | 5087 | 0.065 | -0.1434 | No |
| 76 | CDC42BPA | CDC42 binding protein kinase alpha [Source:HGNC Symbol;Acc:HGNC:1737] | 5097 | 0.065 | -0.1435 | No |
| 77 | EIF2AK2 | eukaryotic translation initiation factor 2 alpha kinase 2 [Source:HGNC Symbol;Acc:HGNC:9437] | 5145 | 0.060 | -0.1465 | No |
| 78 | NEK7 | NIMA related kinase 7 [Source:HGNC Symbol;Acc:HGNC:13386] | 5147 | 0.060 | -0.1460 | No |
| 79 | DYRK2 | dual specificity tyrosine phosphorylation regulated kinase 2 [Source:HGNC Symbol;Acc:HGNC:3093] | 5224 | 0.054 | -0.1514 | No |
| 80 | OBSCN | "obscurin, cytoskeletal calmodulin and titin-interacting RhoGEF [Source:HGNC Symbol;Acc:HGNC:15719]" | 5230 | 0.054 | -0.1512 | No |
| 81 | TLK1 | tousled like kinase 1 [Source:HGNC Symbol;Acc:HGNC:11841] | 5232 | 0.054 | -0.1507 | No |
| 82 | AURKC | aurora kinase C [Source:HGNC Symbol;Acc:HGNC:11391] | 5256 | 0.052 | -0.1520 | No |
| 83 | SIK3 | SIK family kinase 3 [Source:HGNC Symbol;Acc:HGNC:29165] | 5476 | 0.035 | -0.1690 | No |
| 84 | CAMK1G | calcium/calmodulin dependent protein kinase IG [Source:HGNC Symbol;Acc:HGNC:14585] | 5482 | 0.034 | -0.1690 | No |
| 85 | RPS6KB2 | ribosomal protein S6 kinase B2 [Source:HGNC Symbol;Acc:HGNC:10437] | 5496 | 0.033 | -0.1697 | No |
| 86 | MAST1 | microtubule associated serine/threonine kinase 1 [Source:HGNC Symbol;Acc:HGNC:19034] | 5559 | 0.028 | -0.1743 | No |
| 87 | MAP3K19 | mitogen-activated protein kinase kinase kinase 19 [Source:HGNC Symbol;Acc:HGNC:26249] | 5571 | 0.026 | -0.1749 | No |
| 88 | CDKL5 | cyclin dependent kinase like 5 [Source:HGNC Symbol;Acc:HGNC:11411] | 5655 | 0.019 | -0.1813 | No |
| 89 | ERN1 | endoplasmic reticulum to nucleus signaling 1 [Source:HGNC Symbol;Acc:HGNC:3449] | 5658 | 0.019 | -0.1812 | No |
| 90 | MAP3K13 | mitogen-activated protein kinase kinase kinase 13 [Source:HGNC Symbol;Acc:HGNC:6852] | 5728 | 0.013 | -0.1866 | No |
| 91 | CDK19 | cyclin dependent kinase 19 [Source:HGNC Symbol;Acc:HGNC:19338] | 5733 | 0.013 | -0.1868 | No |
| 92 | BMP2K | BMP2 inducible kinase [Source:HGNC Symbol;Acc:HGNC:18041] | 5760 | 0.011 | -0.1887 | No |
| 93 | MKNK1 | MAPK interacting serine/threonine kinase 1 [Source:HGNC Symbol;Acc:HGNC:7110] | 5830 | 0.007 | -0.1941 | No |
| 94 | STK10 | serine/threonine kinase 10 [Source:HGNC Symbol;Acc:HGNC:11388] | 5883 | 0.003 | -0.1982 | No |
| 95 | IKBKB | inhibitor of nuclear factor kappa B kinase subunit beta [Source:HGNC Symbol;Acc:HGNC:5960] | 5914 | 0.001 | -0.2006 | No |
| 96 | PRKCE | protein kinase C epsilon [Source:HGNC Symbol;Acc:HGNC:9401] | 5926 | 0.000 | -0.2014 | No |
| 97 | PLK4 | polo like kinase 4 [Source:HGNC Symbol;Acc:HGNC:11397] | 6041 | -0.008 | -0.2104 | No |
| 98 | MAPKAPK5 | MAPK activated protein kinase 5 [Source:HGNC Symbol;Acc:HGNC:6889] | 6094 | -0.012 | -0.2144 | No |
| 99 | CSNK2A2 | casein kinase 2 alpha 2 [Source:HGNC Symbol;Acc:HGNC:2459] | 6105 | -0.013 | -0.2150 | No |
| 100 | ATM | ATM serine/threonine kinase [Source:HGNC Symbol;Acc:HGNC:795] | 6179 | -0.019 | -0.2206 | No |
| 101 | RAF1 | "Raf-1 proto-oncogene, serine/threonine kinase [Source:HGNC Symbol;Acc:HGNC:9829]" | 6187 | -0.019 | -0.2210 | No |
| 102 | TLK2 | tousled like kinase 2 [Source:HGNC Symbol;Acc:HGNC:11842] | 6190 | -0.020 | -0.2209 | No |
| 103 | BRSK2 | BR serine/threonine kinase 2 [Source:HGNC Symbol;Acc:HGNC:11405] | 6238 | -0.023 | -0.2244 | No |
| 104 | NEK1 | NIMA related kinase 1 [Source:HGNC Symbol;Acc:HGNC:7744] | 6266 | -0.025 | -0.2263 | No |
| 105 | CLK3 | CDC like kinase 3 [Source:HGNC Symbol;Acc:HGNC:2071] | 6417 | -0.037 | -0.2378 | No |
| 106 | NEK2 | NIMA related kinase 2 [Source:HGNC Symbol;Acc:HGNC:7745] | 6422 | -0.037 | -0.2377 | No |
| 107 | MELK | maternal embryonic leucine zipper kinase [Source:HGNC Symbol;Acc:HGNC:16870] | 6424 | -0.037 | -0.2374 | No |
| 108 | PHKG1 | phosphorylase kinase catalytic subunit gamma 1 [Source:HGNC Symbol;Acc:HGNC:8930] | 6451 | -0.039 | -0.2390 | No |
| 109 | PRKCB | protein kinase C beta [Source:HGNC Symbol;Acc:HGNC:9395] | 6453 | -0.039 | -0.2387 | No |
| 110 | SPEG | striated muscle enriched protein kinase [Source:HGNC Symbol;Acc:HGNC:16901] | 6512 | -0.043 | -0.2428 | No |
| 111 | STK38L | serine/threonine kinase 38 like [Source:HGNC Symbol;Acc:HGNC:17848] | 6540 | -0.045 | -0.2445 | No |
| 112 | SGK2 | serum/glucocorticoid regulated kinase 2 [Source:HGNC Symbol;Acc:HGNC:13900] | 6597 | -0.049 | -0.2484 | No |
| 113 | TTK | TTK protein kinase [Source:HGNC Symbol;Acc:HGNC:12401] | 6670 | -0.054 | -0.2535 | No |
| 114 | PRKD3 | protein kinase D3 [Source:HGNC Symbol;Acc:HGNC:9408] | 6717 | -0.057 | -0.2565 | No |
| 115 | MAPK1 | mitogen-activated protein kinase 1 [Source:HGNC Symbol;Acc:HGNC:6871] | 6853 | -0.068 | -0.2665 | No |
| 116 | MAP3K2 | mitogen-activated protein kinase kinase kinase 2 [Source:HGNC Symbol;Acc:HGNC:6854] | 6976 | -0.078 | -0.2753 | No |
| 117 | PRKD2 | protein kinase D2 [Source:HGNC Symbol;Acc:HGNC:17293] | 7011 | -0.081 | -0.2772 | No |
| 118 | STK17A | serine/threonine kinase 17a [Source:HGNC Symbol;Acc:HGNC:11395] | 7016 | -0.081 | -0.2766 | No |
| 119 | MAP3K1 | mitogen-activated protein kinase kinase kinase 1 [Source:HGNC Symbol;Acc:HGNC:6848] | 7066 | -0.085 | -0.2796 | No |
| 120 | RPS6KA3 | ribosomal protein S6 kinase A3 [Source:HGNC Symbol;Acc:HGNC:10432] | 7092 | -0.087 | -0.2806 | No |
| 121 | PRKCH | protein kinase C eta [Source:HGNC Symbol;Acc:HGNC:9403] | 7141 | -0.092 | -0.2834 | No |
| 122 | MAP2K6 | mitogen-activated protein kinase kinase 6 [Source:HGNC Symbol;Acc:HGNC:6846] | 7246 | -0.099 | -0.2906 | No |
| 123 | NLK | nemo like kinase [Source:HGNC Symbol;Acc:HGNC:29858] | 7336 | -0.105 | -0.2966 | No |
| 124 | MINK1 | misshapen like kinase 1 [Source:HGNC Symbol;Acc:HGNC:17565] | 7488 | -0.117 | -0.3073 | No |
| 125 | RIPK4 | receptor interacting serine/threonine kinase 4 [Source:HGNC Symbol;Acc:HGNC:496] | 7505 | -0.119 | -0.3073 | No |
| 126 | CDKL3 | cyclin dependent kinase like 3 [Source:HGNC Symbol;Acc:HGNC:15483] | 7527 | -0.121 | -0.3076 | No |
| 127 | PAK5 | p21 (RAC1) activated kinase 5 [Source:HGNC Symbol;Acc:HGNC:15916] | 7558 | -0.124 | -0.3087 | No |
| 128 | ERN2 | endoplasmic reticulum to nucleus signaling 2 [Source:HGNC Symbol;Acc:HGNC:16942] | 7565 | -0.124 | -0.3078 | No |
| 129 | TESK1 | testis associated actin remodelling kinase 1 [Source:HGNC Symbol;Acc:HGNC:11731] | 7633 | -0.129 | -0.3117 | No |
| 130 | DYRK1A | dual specificity tyrosine phosphorylation regulated kinase 1A [Source:HGNC Symbol;Acc:HGNC:3091] | 7667 | -0.132 | -0.3129 | No |
| 131 | PIK3CA | "phosphatidylinositol-4,5-bisphosphate 3-kinase catalytic subunit alpha [Source:HGNC Symbol;Acc:HGNC:8975]" | 7733 | -0.136 | -0.3166 | No |
| 132 | PAK2 | p21 (RAC1) activated kinase 2 [Source:HGNC Symbol;Acc:HGNC:8591] | 7762 | -0.139 | -0.3173 | No |
| 133 | CAMK1 | calcium/calmodulin dependent protein kinase I [Source:HGNC Symbol;Acc:HGNC:1459] | 7795 | -0.141 | -0.3183 | No |
| 134 | RPS6KC1 | ribosomal protein S6 kinase C1 [Source:HGNC Symbol;Acc:HGNC:10439] | 7856 | -0.145 | -0.3215 | No |
| 135 | DCLK1 | doublecortin like kinase 1 [Source:HGNC Symbol;Acc:HGNC:2700] | 7861 | -0.145 | -0.3203 | No |
| 136 | CDC42BPB | CDC42 binding protein kinase beta [Source:HGNC Symbol;Acc:HGNC:1738] | 7943 | -0.151 | -0.3251 | No |
| 137 | MAP4K5 | mitogen-activated protein kinase kinase kinase kinase 5 [Source:HGNC Symbol;Acc:HGNC:6867] | 7950 | -0.151 | -0.3239 | No |
| 138 | PIK3CG | "phosphatidylinositol-4,5-bisphosphate 3-kinase catalytic subunit gamma [Source:HGNC Symbol;Acc:HGNC:8978]" | 7953 | -0.152 | -0.3224 | No |
| 139 | AKT3 | AKT serine/threonine kinase 3 [Source:HGNC Symbol;Acc:HGNC:393] | 8008 | -0.156 | -0.3250 | No |
| 140 | RPS6KA6 | ribosomal protein S6 kinase A6 [Source:HGNC Symbol;Acc:HGNC:10435] | 8071 | -0.161 | -0.3282 | No |
| 141 | ARAF | "A-Raf proto-oncogene, serine/threonine kinase [Source:HGNC Symbol;Acc:HGNC:646]" | 8092 | -0.163 | -0.3281 | No |
| 142 | MAP3K14 | mitogen-activated protein kinase kinase kinase 14 [Source:HGNC Symbol;Acc:HGNC:6853] | 8100 | -0.163 | -0.3269 | No |
| 143 | MAPK12 | mitogen-activated protein kinase 12 [Source:HGNC Symbol;Acc:HGNC:6874] | 8161 | -0.168 | -0.3298 | No |
| 144 | BRAF | "B-Raf proto-oncogene, serine/threonine kinase [Source:HGNC Symbol;Acc:HGNC:1097]" | 8171 | -0.169 | -0.3287 | No |
| 145 | CDK17 | cyclin dependent kinase 17 [Source:HGNC Symbol;Acc:HGNC:8750] | 8175 | -0.169 | -0.3271 | No |
| 146 | PAK3 | p21 (RAC1) activated kinase 3 [Source:HGNC Symbol;Acc:HGNC:8592] | 8266 | -0.176 | -0.3324 | No |
| 147 | DYRK1B | dual specificity tyrosine phosphorylation regulated kinase 1B [Source:HGNC Symbol;Acc:HGNC:3092] | 8346 | -0.182 | -0.3367 | No |
| 148 | CDK6 | cyclin dependent kinase 6 [Source:HGNC Symbol;Acc:HGNC:1777] | 8393 | -0.185 | -0.3383 | No |
| 149 | RPS6KA2 | ribosomal protein S6 kinase A2 [Source:HGNC Symbol;Acc:HGNC:10431] | 8397 | -0.185 | -0.3366 | No |
| 150 | PRKCG | protein kinase C gamma [Source:HGNC Symbol;Acc:HGNC:9402] | 8582 | -0.200 | -0.3490 | No |
| 151 | DAPK1 | death associated protein kinase 1 [Source:HGNC Symbol;Acc:HGNC:2674] | 8588 | -0.200 | -0.3472 | No |
| 152 | MAP3K4 | mitogen-activated protein kinase kinase kinase 4 [Source:HGNC Symbol;Acc:HGNC:6856] | 8637 | -0.203 | -0.3488 | No |
| 153 | MAST4 | microtubule associated serine/threonine kinase family member 4 [Source:HGNC Symbol;Acc:HGNC:19037] | 8667 | -0.206 | -0.3489 | No |
| 154 | MAPK6 | mitogen-activated protein kinase 6 [Source:HGNC Symbol;Acc:HGNC:6879] | 8710 | -0.210 | -0.3500 | No |
| 155 | DSTYK | dual serine/threonine and tyrosine protein kinase [Source:HGNC Symbol;Acc:HGNC:29043] | 8749 | -0.212 | -0.3507 | Yes |
| 156 | CLK4 | CDC like kinase 4 [Source:HGNC Symbol;Acc:HGNC:13659] | 8760 | -0.213 | -0.3492 | Yes |
| 157 | NEK3 | NIMA related kinase 3 [Source:HGNC Symbol;Acc:HGNC:7746] | 8762 | -0.213 | -0.3470 | Yes |
| 158 | DCAF1 | DDB1 and CUL4 associated factor 1 [Source:HGNC Symbol;Acc:HGNC:30911] | 8765 | -0.213 | -0.3448 | Yes |
| 159 | KSR1 | kinase suppressor of ras 1 [Source:HGNC Symbol;Acc:HGNC:6465] | 8774 | -0.214 | -0.3432 | Yes |
| 160 | CAMK2B | calcium/calmodulin dependent protein kinase II beta [Source:HGNC Symbol;Acc:HGNC:1461] | 8782 | -0.215 | -0.3414 | Yes |
| 161 | DAPK2 | death associated protein kinase 2 [Source:HGNC Symbol;Acc:HGNC:2675] | 8818 | -0.219 | -0.3418 | Yes |
| 162 | PRKAA2 | protein kinase AMP-activated catalytic subunit alpha 2 [Source:HGNC Symbol;Acc:HGNC:9377] | 8864 | -0.222 | -0.3430 | Yes |
| 163 | LRRK1 | leucine rich repeat kinase 1 [Source:HGNC Symbol;Acc:HGNC:18608] | 8881 | -0.223 | -0.3419 | Yes |
| 164 | OXSR1 | oxidative stress responsive kinase 1 [Source:HGNC Symbol;Acc:HGNC:8508] | 8897 | -0.224 | -0.3406 | Yes |
| 165 | TNK2 | tyrosine kinase non receptor 2 [Source:HGNC Symbol;Acc:HGNC:19297] | 8955 | -0.228 | -0.3427 | Yes |
| 166 | MAPK8 | mitogen-activated protein kinase 8 [Source:HGNC Symbol;Acc:HGNC:6881] | 8956 | -0.228 | -0.3402 | Yes |
| 167 | PAK6 | p21 (RAC1) activated kinase 6 [Source:HGNC Symbol;Acc:HGNC:16061] | 9068 | -0.237 | -0.3465 | Yes |
| 168 | PCK1 | phosphoenolpyruvate carboxykinase 1 [Source:HGNC Symbol;Acc:HGNC:8724] | 9100 | -0.239 | -0.3463 | Yes |
| 169 | PRKG2 | protein kinase cGMP-dependent 2 [Source:HGNC Symbol;Acc:HGNC:9416] | 9126 | -0.242 | -0.3457 | Yes |
| 170 | MOS | "MOS proto-oncogene, serine/threonine kinase [Source:HGNC Symbol;Acc:HGNC:7199]" | 9138 | -0.243 | -0.3440 | Yes |
| 171 | TSSK1B | testis specific serine kinase 1B [Source:HGNC Symbol;Acc:HGNC:14968] | 9178 | -0.246 | -0.3444 | Yes |
| 172 | CIITA | class II major histocompatibility complex transactivator [Source:HGNC Symbol;Acc:HGNC:7067] | 9184 | -0.246 | -0.3422 | Yes |
| 173 | NUAK1 | NUAK family kinase 1 [Source:HGNC Symbol;Acc:HGNC:14311] | 9190 | -0.247 | -0.3399 | Yes |
| 174 | STK39 | serine/threonine kinase 39 [Source:HGNC Symbol;Acc:HGNC:17717] | 9206 | -0.248 | -0.3384 | Yes |
| 175 | PRPF4B | pre-mRNA processing factor 4B [Source:HGNC Symbol;Acc:HGNC:17346] | 9210 | -0.249 | -0.3360 | Yes |
| 176 | CDK12 | cyclin dependent kinase 12 [Source:HGNC Symbol;Acc:HGNC:24224] | 9225 | -0.250 | -0.3344 | Yes |
| 177 | MAST3 | microtubule associated serine/threonine kinase 3 [Source:HGNC Symbol;Acc:HGNC:19036] | 9235 | -0.251 | -0.3324 | Yes |
| 178 | STK32B | serine/threonine kinase 32B [Source:HGNC Symbol;Acc:HGNC:14217] | 9258 | -0.252 | -0.3314 | Yes |
| 179 | MAP3K11 | mitogen-activated protein kinase kinase kinase 11 [Source:HGNC Symbol;Acc:HGNC:6850] | 9331 | -0.258 | -0.3343 | Yes |
| 180 | CIT | citron rho-interacting serine/threonine kinase [Source:HGNC Symbol;Acc:HGNC:1985] | 9356 | -0.260 | -0.3334 | Yes |
| 181 | SLK | STE20 like kinase [Source:HGNC Symbol;Acc:HGNC:11088] | 9362 | -0.261 | -0.3310 | Yes |
| 182 | IRAK1 | interleukin 1 receptor associated kinase 1 [Source:HGNC Symbol;Acc:HGNC:6112] | 9377 | -0.262 | -0.3293 | Yes |
| 183 | CSNK1G1 | casein kinase 1 gamma 1 [Source:HGNC Symbol;Acc:HGNC:2454] | 9378 | -0.262 | -0.3265 | Yes |
| 184 | RPS6KA5 | ribosomal protein S6 kinase A5 [Source:HGNC Symbol;Acc:HGNC:10434] | 9406 | -0.265 | -0.3258 | Yes |
| 185 | CDKL2 | cyclin dependent kinase like 2 [Source:HGNC Symbol;Acc:HGNC:1782] | 9453 | -0.269 | -0.3265 | Yes |
| 186 | PLK2 | polo like kinase 2 [Source:HGNC Symbol;Acc:HGNC:19699] | 9482 | -0.273 | -0.3258 | Yes |
| 187 | CDK8 | cyclin dependent kinase 8 [Source:HGNC Symbol;Acc:HGNC:1779] | 9513 | -0.275 | -0.3252 | Yes |
| 188 | MAP4K3 | mitogen-activated protein kinase kinase kinase kinase 3 [Source:HGNC Symbol;Acc:HGNC:6865] | 9520 | -0.276 | -0.3227 | Yes |
| 189 | CDK18 | cyclin dependent kinase 18 [Source:HGNC Symbol;Acc:HGNC:8751] | 9580 | -0.281 | -0.3243 | Yes |
| 190 | MARK4 | microtubule affinity regulating kinase 4 [Source:HGNC Symbol;Acc:HGNC:13538] | 9590 | -0.282 | -0.3220 | Yes |
| 191 | PRKDC | "protein kinase, DNA-activated, catalytic subunit [Source:HGNC Symbol;Acc:HGNC:9413]" | 9632 | -0.286 | -0.3222 | Yes |
| 192 | KALRN | kalirin RhoGEF kinase [Source:HGNC Symbol;Acc:HGNC:4814] | 9649 | -0.287 | -0.3203 | Yes |
| 193 | MARK2 | microtubule affinity regulating kinase 2 [Source:HGNC Symbol;Acc:HGNC:3332] | 9662 | -0.288 | -0.3182 | Yes |
| 194 | PLK3 | polo like kinase 3 [Source:HGNC Symbol;Acc:HGNC:2154] | 9698 | -0.291 | -0.3178 | Yes |
| 195 | MAPK10 | mitogen-activated protein kinase 10 [Source:HGNC Symbol;Acc:HGNC:6872] | 9699 | -0.291 | -0.3147 | Yes |
| 196 | MAP3K9 | mitogen-activated protein kinase kinase kinase 9 [Source:HGNC Symbol;Acc:HGNC:6861] | 9712 | -0.293 | -0.3125 | Yes |
| 197 | LIMK1 | LIM domain kinase 1 [Source:HGNC Symbol;Acc:HGNC:6613] | 9746 | -0.296 | -0.3119 | Yes |
| 198 | CDC7 | cell division cycle 7 [Source:HGNC Symbol;Acc:HGNC:1745] | 9780 | -0.299 | -0.3113 | Yes |
| 199 | ULK1 | unc-51 like autophagy activating kinase 1 [Source:HGNC Symbol;Acc:HGNC:12558] | 9798 | -0.301 | -0.3094 | Yes |
| 200 | LIMK2 | LIM domain kinase 2 [Source:HGNC Symbol;Acc:HGNC:6614] | 9812 | -0.302 | -0.3072 | Yes |
| 201 | STK17B | serine/threonine kinase 17b [Source:HGNC Symbol;Acc:HGNC:11396] | 9825 | -0.303 | -0.3048 | Yes |
| 202 | BCR | BCR activator of RhoGEF and GTPase [Source:HGNC Symbol;Acc:HGNC:1014] | 9830 | -0.304 | -0.3019 | Yes |
| 203 | CDK20 | cyclin dependent kinase 20 [Source:HGNC Symbol;Acc:HGNC:21420] | 9834 | -0.304 | -0.2988 | Yes |
| 204 | STK38 | serine/threonine kinase 38 [Source:HGNC Symbol;Acc:HGNC:17847] | 9912 | -0.313 | -0.3016 | Yes |
| 205 | MAP2K2 | mitogen-activated protein kinase kinase 2 [Source:HGNC Symbol;Acc:HGNC:6842] | 9932 | -0.315 | -0.2997 | Yes |
| 206 | MOK | MOK protein kinase [Source:HGNC Symbol;Acc:HGNC:9833] | 9984 | -0.319 | -0.3003 | Yes |
| 207 | PAK4 | p21 (RAC1) activated kinase 4 [Source:HGNC Symbol;Acc:HGNC:16059] | 10004 | -0.322 | -0.2983 | Yes |
| 208 | EIF2AK3 | eukaryotic translation initiation factor 2 alpha kinase 3 [Source:HGNC Symbol;Acc:HGNC:3255] | 10025 | -0.324 | -0.2964 | Yes |
| 209 | MAPK4 | mitogen-activated protein kinase 4 [Source:HGNC Symbol;Acc:HGNC:6878] | 10042 | -0.325 | -0.2942 | Yes |
| 210 | MAP3K3 | mitogen-activated protein kinase kinase kinase 3 [Source:HGNC Symbol;Acc:HGNC:6855] | 10121 | -0.333 | -0.2967 | Yes |
| 211 | MAP4K4 | mitogen-activated protein kinase kinase kinase kinase 4 [Source:HGNC Symbol;Acc:HGNC:6866] | 10134 | -0.334 | -0.2941 | Yes |
| 212 | SIK2 | salt inducible kinase 2 [Source:HGNC Symbol;Acc:HGNC:21680] | 10189 | -0.339 | -0.2947 | Yes |
| 213 | MAP2K1 | mitogen-activated protein kinase kinase 1 [Source:HGNC Symbol;Acc:HGNC:6840] | 10197 | -0.340 | -0.2916 | Yes |
| 214 | RPS6KB1 | ribosomal protein S6 kinase B1 [Source:HGNC Symbol;Acc:HGNC:10436] | 10224 | -0.343 | -0.2900 | Yes |
| 215 | MAK | male germ cell associated kinase [Source:HGNC Symbol;Acc:HGNC:6816] | 10237 | -0.345 | -0.2872 | Yes |
| 216 | GAK | cyclin G associated kinase [Source:HGNC Symbol;Acc:HGNC:4113] | 10301 | -0.350 | -0.2884 | Yes |
| 217 | MAP3K6 | mitogen-activated protein kinase kinase kinase 6 [Source:HGNC Symbol;Acc:HGNC:6858] | 10317 | -0.353 | -0.2858 | Yes |
| 218 | DMPK | DM1 protein kinase [Source:HGNC Symbol;Acc:HGNC:2933] | 10331 | -0.354 | -0.2830 | Yes |
| 219 | TRIO | trio Rho guanine nucleotide exchange factor [Source:HGNC Symbol;Acc:HGNC:12303] | 10362 | -0.357 | -0.2815 | Yes |
| 220 | HIPK1 | homeodomain interacting protein kinase 1 [Source:HGNC Symbol;Acc:HGNC:19006] | 10391 | -0.360 | -0.2799 | Yes |
| 221 | PRKACG | protein kinase cAMP-activated catalytic subunit gamma [Source:HGNC Symbol;Acc:HGNC:9382] | 10466 | -0.370 | -0.2818 | Yes |
| 222 | SRPK2 | SRSF protein kinase 2 [Source:HGNC Symbol;Acc:HGNC:11306] | 10522 | -0.376 | -0.2821 | Yes |
| 223 | PRKACA | protein kinase cAMP-activated catalytic subunit alpha [Source:HGNC Symbol;Acc:HGNC:9380] | 10611 | -0.384 | -0.2849 | Yes |
| 224 | ROCK2 | Rho associated coiled-coil containing protein kinase 2 [Source:HGNC Symbol;Acc:HGNC:10252] | 10648 | -0.388 | -0.2836 | Yes |
| 225 | STK3 | serine/threonine kinase 3 [Source:HGNC Symbol;Acc:HGNC:11406] | 10656 | -0.388 | -0.2799 | Yes |
| 226 | CDK13 | cyclin dependent kinase 13 [Source:HGNC Symbol;Acc:HGNC:1733] | 10675 | -0.390 | -0.2771 | Yes |
| 227 | MAST2 | microtubule associated serine/threonine kinase 2 [Source:HGNC Symbol;Acc:HGNC:19035] | 10703 | -0.394 | -0.2750 | Yes |
| 228 | TAOK2 | TAO kinase 2 [Source:HGNC Symbol;Acc:HGNC:16835] | 10718 | -0.395 | -0.2719 | Yes |
| 229 | CAMK1D | calcium/calmodulin dependent protein kinase ID [Source:HGNC Symbol;Acc:HGNC:19341] | 10775 | -0.403 | -0.2720 | Yes |
| 230 | WNK1 | WNK lysine deficient protein kinase 1 [Source:HGNC Symbol;Acc:HGNC:14540] | 10777 | -0.403 | -0.2677 | Yes |
| 231 | IRAK4 | interleukin 1 receptor associated kinase 4 [Source:HGNC Symbol;Acc:HGNC:17967] | 10874 | -0.413 | -0.2709 | Yes |
| 232 | MAP3K5 | mitogen-activated protein kinase kinase kinase 5 [Source:HGNC Symbol;Acc:HGNC:6857] | 10923 | -0.421 | -0.2701 | Yes |
| 233 | ALPK3 | alpha kinase 3 [Source:HGNC Symbol;Acc:HGNC:17574] | 10973 | -0.427 | -0.2694 | Yes |
| 234 | MARK1 | microtubule affinity regulating kinase 1 [Source:HGNC Symbol;Acc:HGNC:6896] | 11031 | -0.434 | -0.2693 | Yes |
| 235 | MARK3 | microtubule affinity regulating kinase 3 [Source:HGNC Symbol;Acc:HGNC:6897] | 11063 | -0.440 | -0.2670 | Yes |
| 236 | CDK2 | cyclin dependent kinase 2 [Source:HGNC Symbol;Acc:HGNC:1771] | 11069 | -0.441 | -0.2626 | Yes |
| 237 | CDK9 | cyclin dependent kinase 9 [Source:HGNC Symbol;Acc:HGNC:1780] | 11071 | -0.441 | -0.2579 | Yes |
| 238 | STK4 | serine/threonine kinase 4 [Source:HGNC Symbol;Acc:HGNC:11408] | 11074 | -0.441 | -0.2533 | Yes |
| 239 | TRPM6 | transient receptor potential cation channel subfamily M member 6 [Source:HGNC Symbol;Acc:HGNC:17995] | 11080 | -0.442 | -0.2490 | Yes |
| 240 | CDKL1 | cyclin dependent kinase like 1 [Source:HGNC Symbol;Acc:HGNC:1781] | 11109 | -0.444 | -0.2464 | Yes |
| 241 | STK26 | serine/threonine kinase 26 [Source:HGNC Symbol;Acc:HGNC:18174] | 11152 | -0.451 | -0.2449 | Yes |
| 242 | GSK3B | glycogen synthase kinase 3 beta [Source:HGNC Symbol;Acc:HGNC:4617] | 11157 | -0.451 | -0.2403 | Yes |
| 243 | ATR | ATR serine/threonine kinase [Source:HGNC Symbol;Acc:HGNC:882] | 11231 | -0.461 | -0.2411 | Yes |
| 244 | CSNK1A1 | casein kinase 1 alpha 1 [Source:HGNC Symbol;Acc:HGNC:2451] | 11310 | -0.472 | -0.2422 | Yes |
| 245 | TBK1 | TANK binding kinase 1 [Source:HGNC Symbol;Acc:HGNC:11584] | 11326 | -0.474 | -0.2383 | Yes |
| 246 | MAP3K12 | mitogen-activated protein kinase kinase kinase 12 [Source:HGNC Symbol;Acc:HGNC:6851] | 11344 | -0.478 | -0.2345 | Yes |
| 247 | CLK2 | CDC like kinase 2 [Source:HGNC Symbol;Acc:HGNC:2069] | 11368 | -0.482 | -0.2311 | Yes |
| 248 | PKMYT1 | "protein kinase, membrane associated tyrosine/threonine 1 [Source:HGNC Symbol;Acc:HGNC:29650]" | 11403 | -0.487 | -0.2286 | Yes |
| 249 | STK24 | serine/threonine kinase 24 [Source:HGNC Symbol;Acc:HGNC:11403] | 11409 | -0.488 | -0.2237 | Yes |
| 250 | STK11 | serine/threonine kinase 11 [Source:HGNC Symbol;Acc:HGNC:11389] | 11451 | -0.496 | -0.2216 | Yes |
| 251 | MAP3K20 | mitogen-activated protein kinase kinase kinase 20 [Source:HGNC Symbol;Acc:HGNC:17797] | 11475 | -0.498 | -0.2180 | Yes |
| 252 | CSNK1E | casein kinase 1 epsilon [Source:HGNC Symbol;Acc:HGNC:2453] | 11491 | -0.500 | -0.2138 | Yes |
| 253 | PRKAA1 | protein kinase AMP-activated catalytic subunit alpha 1 [Source:HGNC Symbol;Acc:HGNC:9376] | 11499 | -0.501 | -0.2090 | Yes |
| 254 | MAPK11 | mitogen-activated protein kinase 11 [Source:HGNC Symbol;Acc:HGNC:6873] | 11501 | -0.501 | -0.2037 | Yes |
| 255 | PRKX | protein kinase X-linked [Source:HGNC Symbol;Acc:HGNC:9441] | 11634 | -0.522 | -0.2085 | Yes |
| 256 | AATK | apoptosis associated tyrosine kinase [Source:HGNC Symbol;Acc:HGNC:21] | 11720 | -0.536 | -0.2094 | Yes |
| 257 | MAP3K10 | mitogen-activated protein kinase kinase kinase 10 [Source:HGNC Symbol;Acc:HGNC:6849] | 11753 | -0.541 | -0.2061 | Yes |
| 258 | LATS1 | large tumor suppressor kinase 1 [Source:HGNC Symbol;Acc:HGNC:6514] | 11760 | -0.542 | -0.2008 | Yes |
| 259 | LMTK2 | lemur tyrosine kinase 2 [Source:HGNC Symbol;Acc:HGNC:17880] | 11764 | -0.544 | -0.1951 | Yes |
| 260 | PDPK1 | 3-phosphoinositide dependent protein kinase 1 [Source:HGNC Symbol;Acc:HGNC:8816] | 11839 | -0.557 | -0.1950 | Yes |
| 261 | AAK1 | AP2 associated kinase 1 [Source:HGNC Symbol;Acc:HGNC:19679] | 11842 | -0.558 | -0.1892 | Yes |
| 262 | SRPK1 | SRSF protein kinase 1 [Source:HGNC Symbol;Acc:HGNC:11305] | 11889 | -0.569 | -0.1867 | Yes |
| 263 | CSNK2A1 | casein kinase 2 alpha 1 [Source:HGNC Symbol;Acc:HGNC:2457] | 11895 | -0.570 | -0.1809 | Yes |
| 264 | CDK10 | cyclin dependent kinase 10 [Source:HGNC Symbol;Acc:HGNC:1770] | 11930 | -0.579 | -0.1774 | Yes |
| 265 | CDK11B | cyclin dependent kinase 11B [Source:HGNC Symbol;Acc:HGNC:1729] | 11952 | -0.584 | -0.1727 | Yes |
| 266 | RIPK1 | receptor interacting serine/threonine kinase 1 [Source:HGNC Symbol;Acc:HGNC:10019] | 11953 | -0.584 | -0.1664 | Yes |
| 267 | MAP3K8 | mitogen-activated protein kinase kinase kinase 8 [Source:HGNC Symbol;Acc:HGNC:6860] | 11975 | -0.589 | -0.1617 | Yes |
| 268 | MAP2K3 | mitogen-activated protein kinase kinase 3 [Source:HGNC Symbol;Acc:HGNC:6843] | 12004 | -0.593 | -0.1576 | Yes |
| 269 | ROCK1 | Rho associated coiled-coil containing protein kinase 1 [Source:HGNC Symbol;Acc:HGNC:10251] | 12046 | -0.605 | -0.1543 | Yes |
| 270 | MTOR | mechanistic target of rapamycin kinase [Source:HGNC Symbol;Acc:HGNC:3942] | 12047 | -0.605 | -0.1477 | Yes |
| 271 | TTBK2 | tau tubulin kinase 2 [Source:HGNC Symbol;Acc:HGNC:19141] | 12083 | -0.615 | -0.1439 | Yes |
| 272 | MAPKAPK2 | MAPK activated protein kinase 2 [Source:HGNC Symbol;Acc:HGNC:6887] | 12094 | -0.619 | -0.1380 | Yes |
| 273 | DAPK3 | death associated protein kinase 3 [Source:HGNC Symbol;Acc:HGNC:2676] | 12180 | -0.639 | -0.1379 | Yes |
| 274 | MAP2K7 | mitogen-activated protein kinase kinase 7 [Source:HGNC Symbol;Acc:HGNC:6847] | 12209 | -0.649 | -0.1331 | Yes |
| 275 | MKNK2 | MAPK interacting serine/threonine kinase 2 [Source:HGNC Symbol;Acc:HGNC:7111] | 12236 | -0.655 | -0.1281 | Yes |
| 276 | PKN2 | protein kinase N2 [Source:HGNC Symbol;Acc:HGNC:9406] | 12237 | -0.655 | -0.1210 | Yes |
| 277 | SMG1 | SMG1 nonsense mediated mRNA decay associated PI3K related kinase [Source:HGNC Symbol;Acc:HGNC:30045] | 12268 | -0.668 | -0.1162 | Yes |
| 278 | CDK3 | cyclin dependent kinase 3 [Source:HGNC Symbol;Acc:HGNC:1772] | 12337 | -0.687 | -0.1142 | Yes |
| 279 | PIKFYVE | "phosphoinositide kinase, FYVE-type zinc finger containing [Source:HGNC Symbol;Acc:HGNC:23785]" | 12347 | -0.690 | -0.1074 | Yes |
| 280 | CLK1 | CDC like kinase 1 [Source:HGNC Symbol;Acc:HGNC:2068] | 12354 | -0.693 | -0.1004 | Yes |
| 281 | SGK1 | serum/glucocorticoid regulated kinase 1 [Source:HGNC Symbol;Acc:HGNC:10810] | 12357 | -0.694 | -0.0931 | Yes |
| 282 | DYRK3 | dual specificity tyrosine phosphorylation regulated kinase 3 [Source:HGNC Symbol;Acc:HGNC:3094] | 12379 | -0.702 | -0.0872 | Yes |
| 283 | GSK3A | glycogen synthase kinase 3 alpha [Source:HGNC Symbol;Acc:HGNC:4616] | 12384 | -0.703 | -0.0799 | Yes |
| 284 | HIPK3 | homeodomain interacting protein kinase 3 [Source:HGNC Symbol;Acc:HGNC:4915] | 12404 | -0.707 | -0.0738 | Yes |
| 285 | PRKCI | protein kinase C iota [Source:HGNC Symbol;Acc:HGNC:9404] | 12444 | -0.723 | -0.0691 | Yes |
| 286 | ALPK1 | alpha kinase 1 [Source:HGNC Symbol;Acc:HGNC:20917] | 12481 | -0.741 | -0.0640 | Yes |
| 287 | PIM1 | "Pim-1 proto-oncogene, serine/threonine kinase [Source:HGNC Symbol;Acc:HGNC:8986]" | 12500 | -0.748 | -0.0573 | Yes |
| 288 | PRKY | protein kinase Y-linked (pseudogene) [Source:HGNC Symbol;Acc:HGNC:9444] | 12560 | -0.777 | -0.0536 | Yes |
| 289 | SNRK | SNF related kinase [Source:HGNC Symbol;Acc:HGNC:30598] | 12700 | -0.864 | -0.0553 | Yes |
| 290 | CSNK1G2 | casein kinase 1 gamma 2 [Source:HGNC Symbol;Acc:HGNC:2455] | 12729 | -0.890 | -0.0479 | Yes |
| 291 | TAOK1 | TAO kinase 1 [Source:HGNC Symbol;Acc:HGNC:29259] | 12753 | -0.918 | -0.0398 | Yes |
| 292 | HIPK2 | homeodomain interacting protein kinase 2 [Source:HGNC Symbol;Acc:HGNC:14402] | 12842 | -1.045 | -0.0356 | Yes |
| 293 | SIK1 | salt inducible kinase 1 [Source:HGNC Symbol;Acc:HGNC:11142] | 12863 | -1.104 | -0.0252 | Yes |
| 294 | TAF1 | TATA-box binding protein associated factor 1 [Source:HGNC Symbol;Acc:HGNC:11535] | 12881 | -1.192 | -0.0137 | Yes |
| 295 | AKT2 | AKT serine/threonine kinase 2 [Source:HGNC Symbol;Acc:HGNC:392] | 12896 | -1.404 | 0.0003 | Yes |
Table: GSEA details [plain text format]

  

Fig 2: GOMF\_PROTEIN\_SERINE\_KINASE\_ACTIVITY      
 Blue-Pink O' Gram in the Space of the Analyzed GeneSet

  

Fig 3: GOMF\_PROTEIN\_SERINE\_KINASE\_ACTIVITY: Random ES distribution      
 Gene set null distribution of ES for **GOMF\_PROTEIN\_SERINE\_KINASE\_ACTIVITY**
